# Development of the glioma imaging complexity score (GICS): a volumetric MRI-based stratification framework

**DOI:** 10.3389/fsurg.2026.1872271

**Published:** 2026-06-29

**Authors:** Alex Ofori, Guozhu Sun

**Affiliations:** Department of Neurosurgery, The Second Hospital of Hebei Medical University, Shijiazhuang, China

**Keywords:** BRATS, GICS, glioma, imaging complexity, MRI, neuronavigation, quantitative imaging, radiomics

## Abstract

**Background:**

Quantitative MRI analysis and radiomics-based imaging assessment have increasingly contributed to neuro-oncological research by improving reproducibility and minimizing dependence on subjective radiological interpretation. Despite advancements in imaging analysis and visualization support technologies, standardized quantitative frameworks for classifying glioma MRI-derived complexity remain limited.

**Objective:**

To establish a quantitative framework based on MRI data for stratifying the complexity of glioma imaging. This framework utilizes standardized imaging features derived from segmentation, obtained through the Brain Tumor Segmentation (BraTS) initiative.

**Methods:**

A retrospective quantitative MRI analysis was performed using segmentation datasets from 1,251 glioma cases obtained from the publicly available BraTS repository. Quantitative imaging variables, including total tumor volume, enhancing tumor volume, edema volume, necrotic/non-enhancing core volume, and maximum tumor diameter, were extracted using voxel-based segmentation methods. Cases were ranked according to total tumor volume and subsequently categorized into low-, moderate-, and high-complexity groups corresponding to GICS-1, GICS-2, and GICS-3 classifications. Statistical analysis included ANOVA, Kruskal–Wallis testing, and Pearson/Spearman correlation analysis.

**Results:**

Tertile stratification produced three evenly distributed groups, each comprising 417 cases. The mean total tumor volume increased across GICS categories, from 35.40 ± 15.84 cm^3^ in GICS-1 to 162.62 ± 34.96 cm^3^ in GICS-3 (*p* < 0.001). An escalation in the GICS category was also correlated with a greater edema burden, increased maximum tumor diameter, and larger enhancing and necrotic components within the tumor. Notably, robust positive correlations were observed between total tumor volume and maximum tumor diameter (Pearson *r* = 0.764; *p* < 0.001) as well as between total tumor volume and edema volume (Pearson *r* = 0.861; *p* < 0.001). Overall, higher GICS categories demonstrated consistent quantitative differences across segmentation-derived imaging variables.

**Conclusion:**

The GICS framework represents a preliminary quantitative MRI stratification model constructed using standardized segmentation datasets. The results indicate that segmentation-derived MRI variables can be organized into reproducible stratification groups, thereby laying the groundwork for prospective studies on imaging-based complexity assessment and exploratory visualization-support approaches in glioma surgery.

## Introduction

Gliomas remain among the most challenging tumors encountered in neurosurgical practice because of their infiltrative growth, variable morphology, and frequent involvement of critical neuroanatomical structures. Surgical resection continues to play a major role in treatment, particularly in high-grade gliomas, where a greater extent of resection has been associated with improved progression-free and overall survival (1, 2). However, achieving maximal safe resection remains difficult because surgical planning must account for tumor size, edema burden, anatomical distortion, infiltrative spread, and proximity to eloquent cortex and deep white matter pathways.

MRI is central to preoperative glioma evaluation and operative planning. Conventional MRI allows assessment of tumor enhancement, edema, necrosis, mass effect, and anatomical relationships with surrounding brain structures. Nevertheless, interpretation of these imaging features remains partly subjective and may vary between observers depending on radiological and surgical experience. Large or infiltrative tumors frequently distort normal anatomy, compress ventricular structures, and displace surrounding white matter tracts, making it difficult to consistently estimate MRI-derived complexity using routine qualitative MRI review alone.

Another important limitation is the absence of widely accepted quantitative systems capable of organizing glioma cases according to reproducible imaging characteristics. In daily neurosurgical practice, operative complexity is often estimated from a combination of subjective imaging impressions, including lesion size, anatomical location, extent of edema, cortical involvement, and expected distortion of the surgical corridor. Although experienced neurosurgeons intuitively integrate these factors into surgical planning, objective MRI-based stratification methods remain relatively limited.

- The statistical methodology used in this study is summarized in Table 1.
- GICS classification thresholds are presented in Table 2.
- Quantitative MRI-derived tumor characteristics are summarized in Table 3.
- A conceptual comparison with previously reported glioma classification systems is presented in Table 4.
- The radiographic interpretation and neuro-oncological relevance of MRI-derived variables are summarized in Table 5.

**Table 1:** Summary of statistical methods applied during development of the glioma imaging complexity score (GICS) framework.

Variable/Analysis	Data Type	Distribution Assessment	Statistical Test Applied	Purpose of Analysis
Total tumor volume	Continuous volumetric variable	Non-parametric distribution observed across tertiles	Kruskal–Wallis test	Comparison of volumetric burden across GICS-defined complexity categories
Enhancing tumor volume	Continuous volumetric variable	Non-normal distribution with heterogeneous variance	Kruskal–Wallis test	Evaluation of progressive enhancing tumor burden across GICS groups
Edema volume	Continuous volumetric variable	Non-parametric distribution	Kruskal–Wallis test	Assessment of edema progression across radiographic complexity categories
Necrotic/non-enhancing core volume	Continuous volumetric variable	Non-normal distribution with outlier variability	Kruskal–Wallis test	Comparison of necrotic tumor burden across GICS categories
Maximum tumor diameter	Continuous dimensional variable	Approximately normal distribution	One-way ANOVA	Comparison of tumor spatial extent across GICS-defined groups
Correlation between total tumor volume and edema volume	Continuous variables	Linear and monotonic relationship assessment	Pearson correlation and Spearman rank correlation	Evaluation of associations between tumor burden and edema expansion
Correlation between total tumor volume and enhancing tumor volume	Continuous variables	Linear and monotonic relationship assessment	Pearson correlation and Spearman rank correlation	Assessment of enhancing tumor progression relative to total tumor burden
Correlation between total tumor volume and necrotic core volume	Continuous variables	Linear and monotonic relationship assessment	Pearson correlation and Spearman rank correlation	Evaluation of necrotic transformation relative to tumor burden
Correlation between total tumor volume and maximum tumor diameter	Continuous variables	Linear and monotonic relationship assessment	Pearson correlation and Spearman rank correlation	Assessment of spatial anatomical expansion relative to volumetric tumor burden
Statistical significance threshold	—	—	Two-tailed *p*-value < 0.05	Determination of statistical significance across all analyses

The table summarizes statistical approaches used to evaluate quantitative MRI-derived variables across MRI-derived GICS categories. Statistical methodology was selected based on the distributional characteristics of the volumetric imaging variables. Non-parametric tests were used for heterogeneous volumetric measurements with non-normal distributions, whereas parametric tests were used for approximately normally distributed continuous variables.

**Table 2:** Definition of MRI-derived GICS stratification categories based on total tumor volume tertiles.

Complexity group	n	Total volume threshold (cm^3^)	Interpretation
GICS-1 (Low)	417	2.81–63.20	Lowest tertile
GICS-2 (Moderate)	417	63.56–118.35	Middle tertile
GICS-3 (High)	417	118.39–361.78	Highest tertile

Thresholds represent tertile boundaries derived from total tumor volume measurements across the study cohort.

**Table 3:** Quantitative MRI-derived tumor characteristics across GICS-defined imaging complexity categories.

Feature	GICS-1 (Low) (*n* = 417)	GICS-2 (Moderate) (*n* = 417)	GICS-3 (High) (*n* = 417)	Kruskal–Wallis *p*-value
Total tumor volume (cm^3^)	35.40 ± 15.84; 35.13 (22.66–48.05)	89.88 ± 15.19; 89.34 (76.79–102.25)	162.62 ± 34.96; 155.03 (136.83–181.58)	<0.001
Enhancing tumor volume (cm^3^)	8.92 ± 7.56; 6.88 (2.83–12.72)	21.47 ± 13.56; 19.88 (11.74–29.52)	33.95 ± 20.61; 32.17 (18.58–46.43)	<0.001
Edema volume (cm^3^)	22.35 ± 13.24; 20.96 (11.68–31.39)	55.03 ± 21.43; 55.21 (39.34–70.39)	103.27 ± 37.55; 101.90 (76.50–126.65)	<0.001
Necrotic/non-enhancing core volume (cm^3^)	4.13 ± 6.10; 1.79 (0.33–5.37)	13.38 ± 14.53; 8.61 (3.47–18.87)	25.41 ± 28.37; 17.62 (7.27–31.60)	<0.001
Maximum tumor diameter (mm)	94.78 ± 23.57; 93.80 (80.59–107.50)	124.79 ± 19.10; 120.07 (111.85–133.37)	146.53 ± 17.59; 143.70 (135.13–154.54)	<0.001

Values are presented as mean ± standard deviation and median (interquartile range).

**Table 4:** Conceptual comparison between the glioma imaging complexity score (GICS) framework and previously reported glioma classification approaches.

Classification System	Primary Basis of Classification	Quantitative Volumetric Analysis	Operative/Anatomical Variables Included	Validation Approach	Primary Intended Application
Ganau et al. (3)	Surgical complexity assessment based on anatomical relationships	Limited	Yes — eloquent cortex, ventricular involvement, vascular anatomy, white matter tract proximity	Clinical surgical correlation	Prediction of operative difficulty and postoperative outcome
Marcus et al. (4)	MRI-based preoperative grading system	Partial	Partial — anatomical MRI characteristics associated with surgical outcome	Preliminary clinical validation	Prediction of glioblastoma surgical outcomes
Conventional qualitative MRI interpretation	Subjective radiological assessment	No	Variable and observer-dependent	Routine clinical interpretation	General radiological evaluation and operative planning
Radiomics-based computational imaging approaches	High-dimensional computational feature extraction	Yes	Typically limited or indirect	Computational and machine-learning validation	Molecular prediction, survival estimation, imaging biomarker discovery
Glioma Imaging Complexity Score (GICS) — Present Study	MRI-derived volumetric stratification using segmentation-based computational analysis	Yes	No — operative anatomy, tractography, vascular relationships, and intraoperative variables intentionally excluded	Internal computational imaging analysis using harmonized BraTS datasets	Quantitative MRI-derived glioma stratification and exploratory computational neuro-oncology research

The table compares the proposed MRI-based GICS framework with representative anatomical, operative, qualitative imaging, and radiomics-oriented glioma classification systems reported in the literature. Comparison domains include primary classification methodology, incorporation of quantitative volumetric analysis, inclusion of operative anatomical variables, validation strategy, and intended clinical or computational application.

**Table 5:** Radiographic and neuro-oncological interpretation of MRI-derived variables included within the GICS framework.

MRI-Derived Variable	Imaging Source/Segmentation Basis	Radiographic Interpretation	Potential Neuro-Oncological Relevance
Total tumor volume	Combined segmented pathological tumor compartments	Overall tumor burden and spatial anatomical occupation	May reflect increasing mass effect, operative exposure requirements, and anatomical distortion
Enhancing tumor volume	Contrast-enhanced T1-weighted MRI (T1CE)	Active contrast-enhancing tumor component	May reflect biologically active tumor regions and blood–brain barrier disruption
Edema volume	FLAIR hyperintense peritumoral regions	Peritumoral tissue disruption and infiltrative edema burden	May contribute to regional anatomical distortion, neurovascular displacement, and operative planning challenges
Necrotic/non-enhancing core volume	Segmented necrotic and non-enhancing tumor regions	Intratumoral necrosis and heterogeneous tumor degeneration	Frequently associated with aggressive tumor biology and radiographic heterogeneity
Maximum tumor diameter	Three-dimensional Euclidean distance analysis of segmented tumor masks	Spatial anatomical extent of glioma involvement	May reflect infiltrative expansion and increasing spatial anatomical involvement
Segmentation-derived volumetric heterogeneity	Combined analysis of all tumor subregions	Internal radiographic heterogeneity across tumor compartments	Supports computational characterization of glioma imaging heterogeneity
Voxel-based MRI quantification	Standardized BraTS preprocessing and segmentation workflow	Objective computational imaging measurement	Improves reproducibility and reduces observer-dependent radiological variability
Multi-parametric MRI integration	T1, T1CE, T2, and FLAIR MRI sequences	Integrated characterization of glioma morphology and tissue composition	Facilitates harmonized radiomics-oriented computational neuroimaging analysis

The table summarizes the imaging source, radiographic interpretation, and potential neuro-oncological relevance of MRI-derived variables analyzed during development of the GICS model.

Several previous studies have explored imaging and anatomical grading systems for glioma surgery. Ganau et al. proposed a classification system incorporating eloquent cortical involvement, ventricular relationships, vascular anatomy, and proximity to white matter tracts in high-grade glioma surgery (3). Marcus et al. also developed a preoperative MRI grading model to predict surgical outcomes in patients with glioblastoma using anatomical imaging features (4). These studies emphasized the relevance of imaging characteristics for operative planning, although many currently available systems remain partly qualitative or institution-specific.

At the same time, advances in radiomics and quantitative imaging analysis have enabled the extraction of imaging biomarkers from large, standardized MRI datasets. The Brain Tumor Segmentation (BraTS) initiative has contributed substantially to this field by providing standardized, multi-institutional imaging datasets, expert-annotated segmentation masks, and standardized preprocessing pipelines (5, 6). These datasets enable reproducible voxel-based analysis of tumor burden, edema distribution, enhancing tumor components, necrotic regions, and spatial extent of tumors across large glioma cohorts.

Modern neurosurgical workflows have also increasingly incorporated advanced visualization technologies, including three-dimensional reconstruction, neuronavigation, mixed reality, and augmented reality systems designed to improve spatial understanding of distorted neuroanatomy (11–18). Despite these technological developments, standardized MRI-based stratification systems capable of organizing the complexity of glioma imaging remain limited.

The present study therefore introduces the Glioma Imaging Complexity Score (GICS), a quantitative glioma stratification framework developed using segmentation-derived imaging features from the BraTS dataset. Variables analyzed included total tumor volume, edema volume, enhancing tumor volume, necrotic/non-enhancing core volume, and maximum tumor diameter.

The present investigation was designed as an exploratory imaging analysis rather than a validated operative grading system. The framework does not incorporate intraoperative findings, tractography, vascular imaging, functional MRI, postoperative outcomes, or prospective clinical validation. Accordingly, GICS should be interpreted as an imaging-based stratification model rather than a direct predictor of surgical difficulty or clinical outcome.

## Materials and methods

### Study design

A retrospective quantitative MRI analysis was performed using publicly available glioma MRI segmentation datasets obtained from the Brain Tumor Segmentation (BraTS) initiative. The primary objective of the study was to determine whether quantitative MRI-derived imaging variables could be organized into reproducible MRI-derived stratification groups using a standardized computational framework.

The investigation was designed as a quantitative imaging study rather than a prospective clinical validation study. No patient recruitment, clinical intervention, or longitudinal outcome analysis was performed. The study focused specifically on quantitative MRI features extracted from standardized imaging datasets and did not incorporate intraoperative findings, postoperative neurological outcomes, molecular tumor profiling, or survival analysis.

Because the investigation utilized fully anonymized publicly available imaging data, no direct patient interaction occurred during the study.

### Data source and imaging dataset

MRI datasets were obtained from the Brain Tumor Segmentation (BraTS) initiative, an internationally recognized multicenter neuroimaging consortium developed to support computational glioma segmentation research and radiomics-based imaging analysis (5, 6).

The BraTS initiative aggregates MRI datasets from multiple institutions and scanner platforms and provides standardized preprocessing pipelines together with expert-annotated tumor segmentation masks.

The use of a multicenter imaging repository was considered important because glioma MRI characteristics may vary considerably across institutions, acquisition protocols, magnetic field strengths, and scanner manufacturers. To minimize inter-scanner variability and improve reproducibility across datasets, the BraTS consortium applies standardized preprocessing procedures prior to public dataset release.

The MRI dataset included multi-parametric imaging sequences routinely used in neuro-oncological evaluation, including:

- T1-weighted imaging,
- Contrast-enhanced T1-weighted imaging (T1CE),
- T2-weighted imaging,
- Fluid-attenuated inversion recovery (FLAIR) imaging.

Before inclusion in the BraTS repository, MRI datasets underwent standardized preprocessing, including skull stripping, spatial co-registration across imaging modalities, intensity normalization, and resampling to a unified anatomical space. These preprocessing steps were designed to reduce technical variability across imaging centers and to enable more reliable computational analysis across large glioma cohorts.

The BraTS consortium additionally provides expert-annotated segmentation masks for major glioma subregions, including:

- Enhancing tumor,
- Peritumoral edema,
- Necrotic/non-enhancing tumor core.

These segmentation methodologies have undergone extensive validation in prior radiomics and quantitative MRI research and are widely utilized within the neuro-oncology imaging literature (5, 6). The availability of standardized segmentation datasets enabled reproducible extraction of quantitative MRI variables while reducing observer-dependent variability associated with manual segmentation procedures.

### MRI preprocessing and segmentation analysis

The current investigation used annotated tumor masks from the BraTS repository rather than performing manual *de novo* segmentation. This approach was selected to improve reproducibility and maintain consistency across the large imaging cohort included in the analysis.

BraTS preprocessing pipelines included:

- Skull stripping,
- Intensity normalization,
- Multimodal spatial co-registration,
- Isotropic resampling into a common anatomical reference space.

These procedures reduce variability related to scanner acquisition protocols and improve consistency in voxel-level computational analysis across heterogeneous MRI datasets.

Segmentation masks provided within the repository delineated distinct pathological tumor compartments, including enhancing tumor regions, edema, and necrotic/non-enhancing tumor core tissue. These segmented regions formed the basis for subsequent quantitative volumetric analysis and MRI-based stratification.

**Figure 1:**
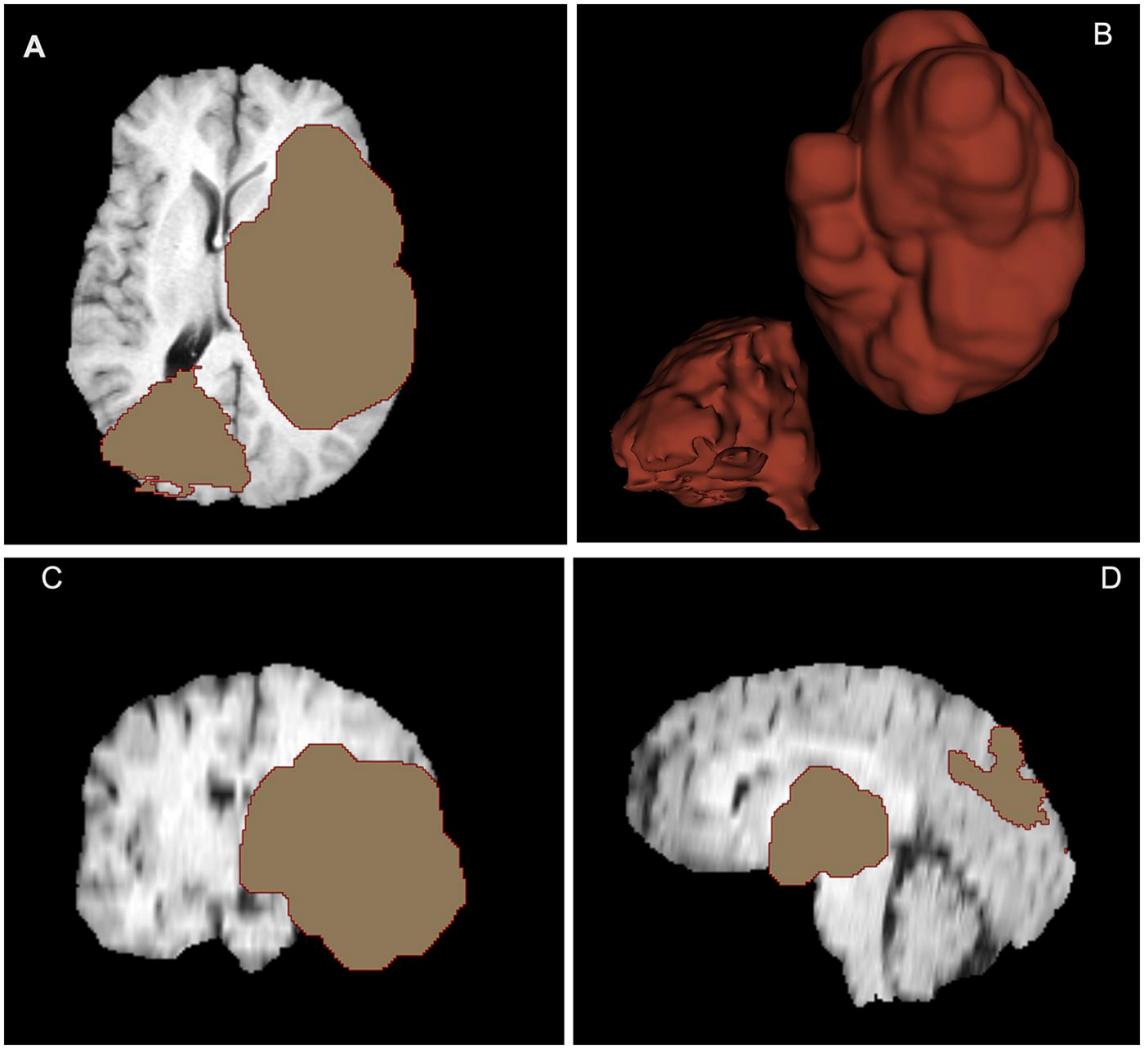
Representative MRI segmentation and three-dimensional volumetric reconstruction workflow used in the development of the glioma imaging complexity score (GICS). **(A)** Axial MRI slice demonstrating tumor segmentation. **(B)** Three-dimensional volumetric reconstruction of the segmented tumor. **(C)** Sagittal view of the segmented lesion. **(D)** Coronal view demonstrating spatial tumor extent. The workflow integrates standardized BraTS preprocessing and voxel-based volumetric analysis to support reproducible characterization of tumor burden and radiographic heterogeneity while reducing observer-dependent variability associated with qualitative MRI assessment.

Representative segmentation and volumetric reconstruction workflows are illustrated in Figure 1.

Quantitative MRI-derived variables were extracted using voxel-based volumetric analysis of segmentation masks. Imaging features selected for analysis were chosen based on their known relevance to glioma morphology, anatomical burden, radiographic heterogeneity, and spatial tumor involvement within neuro-oncological imaging research.

The following variables were extracted:

- Total tumor volume,
- Enhancing tumor volume,
- Edema volume,
- Necrotic/non-enhancing core volume,
- Maximum tumor diameter.

Total tumor volume represented the cumulative pathological tumor burden derived from all segmented tumor compartments. Tumor volume enhancement was calculated from contrast-enhancing regions identified on T1CE imaging, whereas edema volume was derived primarily from FLAIR hyperintense peritumoral regions. Necrotic/non-enhancing tumor core volume was calculated using the corresponding annotated segmentation masks provided within the BraTS dataset.

Volumetric calculations were performed using voxel-count-based computational analysis. In voxel-based imaging analysis, each voxel represents a three-dimensional imaging unit corresponding to a defined volume of tissue within the MRI dataset. Segmented tumor regions were quantified by counting the total number of voxels in each tumor compartment.

Voxel dimensions provided in the imaging metadata were subsequently used to convert measurements from cubic millimeters (mm^3^) to cubic centimeters (cm^3^) to improve interpretability and facilitate comparison with prior neuro-oncological volumetric studies.

Because the datasets underwent isotropic normalization during preprocessing, voxel dimensions remained standardized across cases, thereby improving consistency and reproducibility in volumetric analysis. Compared with conventional two-dimensional radiographic measurements, voxel-based volumetric analysis provides a more comprehensive representation of irregular glioma morphology and infiltrative tumor extension.

### Maximum tumor diameter analysis

Maximum tumor diameter was calculated using three-dimensional Euclidean distance measurements derived from segmented tumor masks. This method enabled orientation-independent estimation of tumor extent and spatial anatomical involvement.

Unlike conventional axial diameter measurements obtained from a single imaging plane, three-dimensional voxel-based analysis permits more accurate characterization of irregular and infiltrative glioma morphology. Maximum tumor diameter was expressed in millimeters (mm) for all analyses.

### Operational definition of radiographic complexity

In the present investigation, imaging complexity was operationally defined as a relative estimate of MRI-derived tumor burden, anatomical distortion, edema distribution, and radiographic heterogeneity, as observed in quantitative volumetric analysis.

Importantly, the proposed GICS model was intentionally developed as an imaging-based organizational framework rather than a validated operative grading system. Several clinically important neurosurgical variables were therefore not incorporated into the current analysis, including:

- Eloquent cortical involvement,
- Diffusion tensor tractography,
- Vascular anatomy,
- White matter tract displacement,
- Ventricular invasion,
- Functional MRI characteristics,
- Intraoperative findings,
- Postoperative neurological outcomes.

Accordingly, the proposed classification system should be interpreted as an imaging stratification framework rather than a direct predictor of surgical complexity or operative outcome.

### Development of the glioma imaging complexity score (GICS)

Cases were ranked according to total tumor volume and subsequently stratified into tertiles corresponding to increasing MRI-derived complexity categories:

- GICS-1,
- GICS-2,
- GICS-3.

Tertile-based stratification was selected to generate balanced statistical group distributions while preserving progressive volumetric separation across the cohort. This approach was additionally chosen to avoid arbitrarily selected threshold values that may not generalize consistently across heterogeneous imaging populations.

Because the present investigation was designed as an exploratory computational imaging study rather than a validated prognostic model, tertile stratification provided a simple and reproducible framework for evaluating progressive relationships between tumor burden and associated MRI-derived variables.

The balanced distribution generated by tertile classification also improved comparative statistical analysis across groups by maintaining similar sample sizes within each category.

**Figure 2:**
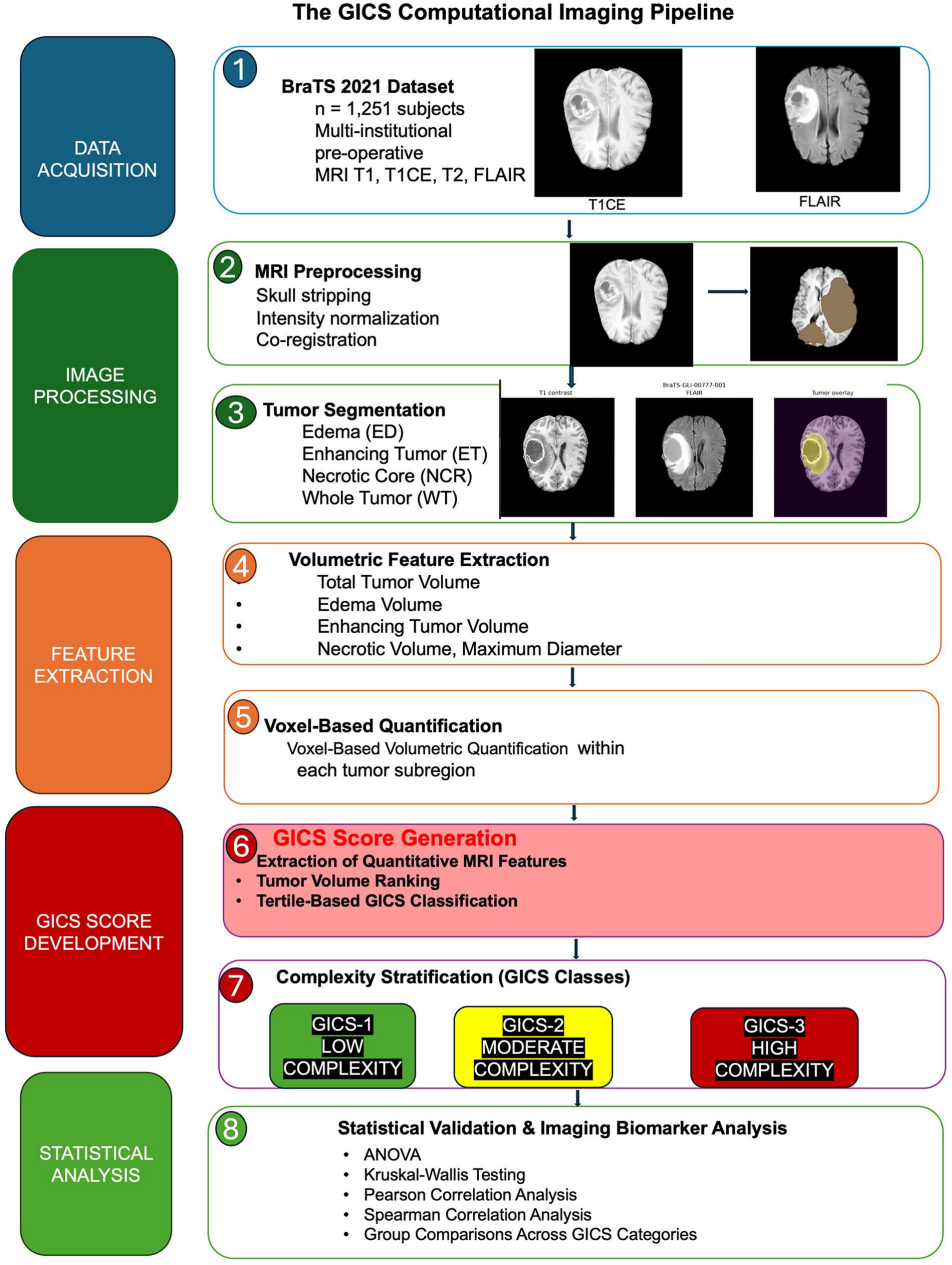
Computational imaging workflow underlying development of the glioma imaging complexity score (GICS).

Additional MRI-derived variables were subsequently evaluated across GICS-defined groups to assess whether increasing tumor burden was associated with coordinated radiographic variation. The overall computational workflow underlying the development of the GICS framework is illustrated in Figure 2.

The workflow illustrates MRI preprocessing, tumor segmentation, volumetric feature extraction, voxel-based quantification, tertile-based classification, and statistical analysis. Quantitative imaging variables extracted from segmentation masks included total tumor volume, edema volume, enhancing tumor volume, necrotic/non-enhancing tumor core volume, and maximum tumor diameter. Cases were subsequently stratified into tertile-based GICS categories (GICS-1, GICS-2, and GICS-3).

### Statistical analysis

Continuous variables were summarized as mean ± standard deviation, with median and interquartile range where appropriate. Distributional characteristics of MRI-derived variables were evaluated prior to comparative statistical analysis.

Because several volumetric variables demonstrated non-normal distributions and heterogeneous variance across groups, non-parametric statistical methods were incorporated to improve analytical robustness. Comparisons across GICS-defined categories were therefore performed using Kruskal–Wallis testing for non-parametric variables and one-way analysis of variance (ANOVA) for approximately normally distributed continuous variables.

The combined use of parametric and non-parametric methods was intended to improve statistical reliability across heterogeneous imaging-derived measurements.

Correlation analyses were additionally performed to evaluate relationships between total tumor volume and other MRI-derived variables. Pearson correlation coefficients were used to assess linear relationships between continuous variables, whereas Spearman rank correlation analysis was performed to evaluate monotonic non-parametric associations that may be less sensitive to outliers or non-normal distributions.

Comparable findings across both Pearson and Spearman analyses supported the stability of the observed imaging relationships.

Correlation strength was interpreted using conventional thresholds:

- Weak correlation: <0.3,
- Moderate correlation: 0.3–0.7,
- Strong correlation: >0.7.

All statistical analyses were two-tailed, and statistical significance was defined as *p* < 0.05.

Combined use of Pearson and Spearman correlation analyses was performed to evaluate the robustness and consistency of observed relationships between total tumor burden and associated MRI-derived imaging features.

### Ethical considerations and data availability

The study utilized exclusively anonymized publicly available MRI datasets obtained from the BraTS initiative. No direct patient interaction, intervention, or identifiable patient information was involved in the current investigation. Accordingly, separate institutional ethical approval and informed consent were not required for this retrospective imaging analysis in accordance with publicly available dataset usage policies.

Publicly available datasets analyzed in this study are accessible through the Brain Tumor Segmentation (BraTS) initiative and associated repositories (5, 6).

## Results

A total of 1,251 glioma cases were included in the computational MRI analysis. Following rank-based tertile stratification according to total tumor volume, three balanced MRI-derived groups were generated, comprising 417 cases each within the GICS-1, GICS-2, and GICS-3 categories.

Cases were stratified into low-, moderate-, and high-complexity categories according to segmented total tumor volume thresholds.

The tertile-based approach produced graded volumetric separation while maintaining equivalent sample sizes across groups for comparative statistical analysis.

Mean total tumor volume increased substantially across GICS categories, rising from 35.40 ± 15.84 cm^3^ in GICS-1 to 89.88 ± 15.19 cm^3^ in GICS-2 and 162.62 ± 34.96 cm^3^ in GICS-3 (*p* < 0.001).

Boxplot analysis demonstrates progressive volumetric separation between GICS-1, GICS-2, and GICS-3 categories. Increasing GICS classification was associated with progressively greater tumor burden, supporting the validity of the tertile-based MRI stratification framework.

Higher GICS categories were associated with greater edema burden, larger enhancing tumor volume, increased necrotic/non-enhancing core volume, and broader spatial anatomical involvement, as reflected by maximum tumor diameter measurements.

Mean maximum tumor diameter increased from 94.78 ± 23.57 mm in GICS-1 to 124.79 ± 19.10 mm in GICS-2 and 146.53 ± 17.59 mm in GICS-3. Similarly, edema volume increased from 22.35 ± 13.24 cm^3^ in GICS-1 to 103.27 ± 37.55 cm^3^ in GICS-3.

Quantitative comparison across GICS categories demonstrated coordinated increases across multiple MRI-derived variables, including edema burden, enhancing tumor volume, necrotic/non-enhancing core volume, and maximum tumor diameter.

Collectively, the data suggest that greater tumor burden is associated with broader anatomical involvement and increasing radiographic heterogeneity on quantitative MRI analysis.

Higher GICS categories were associated with progressively larger maximum tumor diameters. Three-dimensional voxel-based diameter measurements demonstrated increasing spatial anatomical involvement across the stratified glioma cohort.

Correlation analysis demonstrated strong positive associations between total tumor volume and edema volume (Pearson *r* = 0.861; Spearman rho = 0.864; *p* < 0.001), as well as between total tumor volume and maximum tumor diameter (Pearson *r* = 0.764; Spearman rho = 0.799; *p* < 0.001).

Moderate positive correlations were observed between total tumor volume and enhancing tumor volume (Pearson *r* = 0.591; *p* < 0.001) and between total tumor volume and necrotic/non-enhancing core volume (Pearson *r* = 0.497; *p* < 0.001).

Both Pearson and Spearman analyses demonstrated comparable findings, supporting the stability of the observed imaging relationships across the dataset.

**Figure 3:**
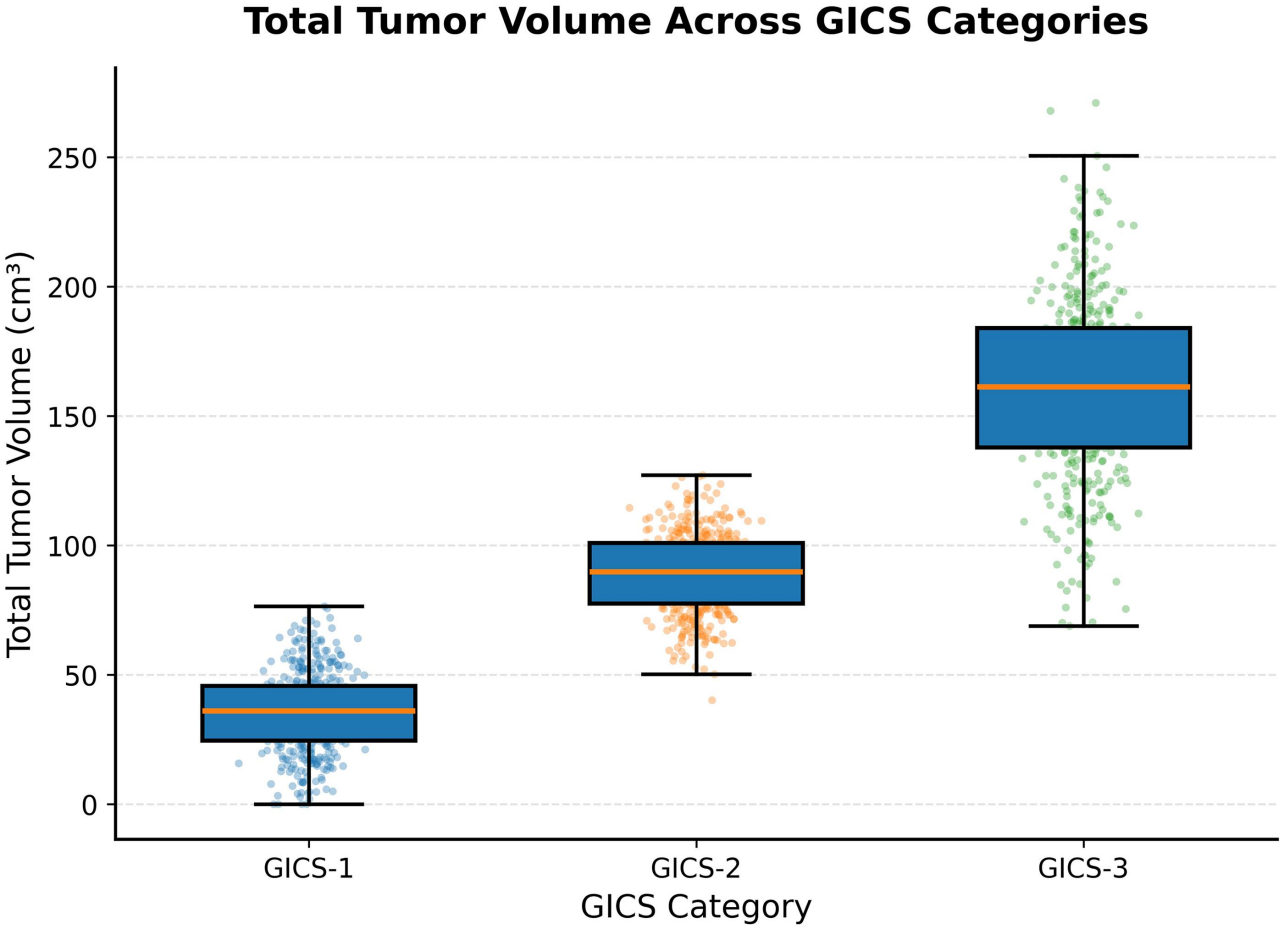
Distribution of total tumor volume across GICS-defined categories.

**Figure 4:**
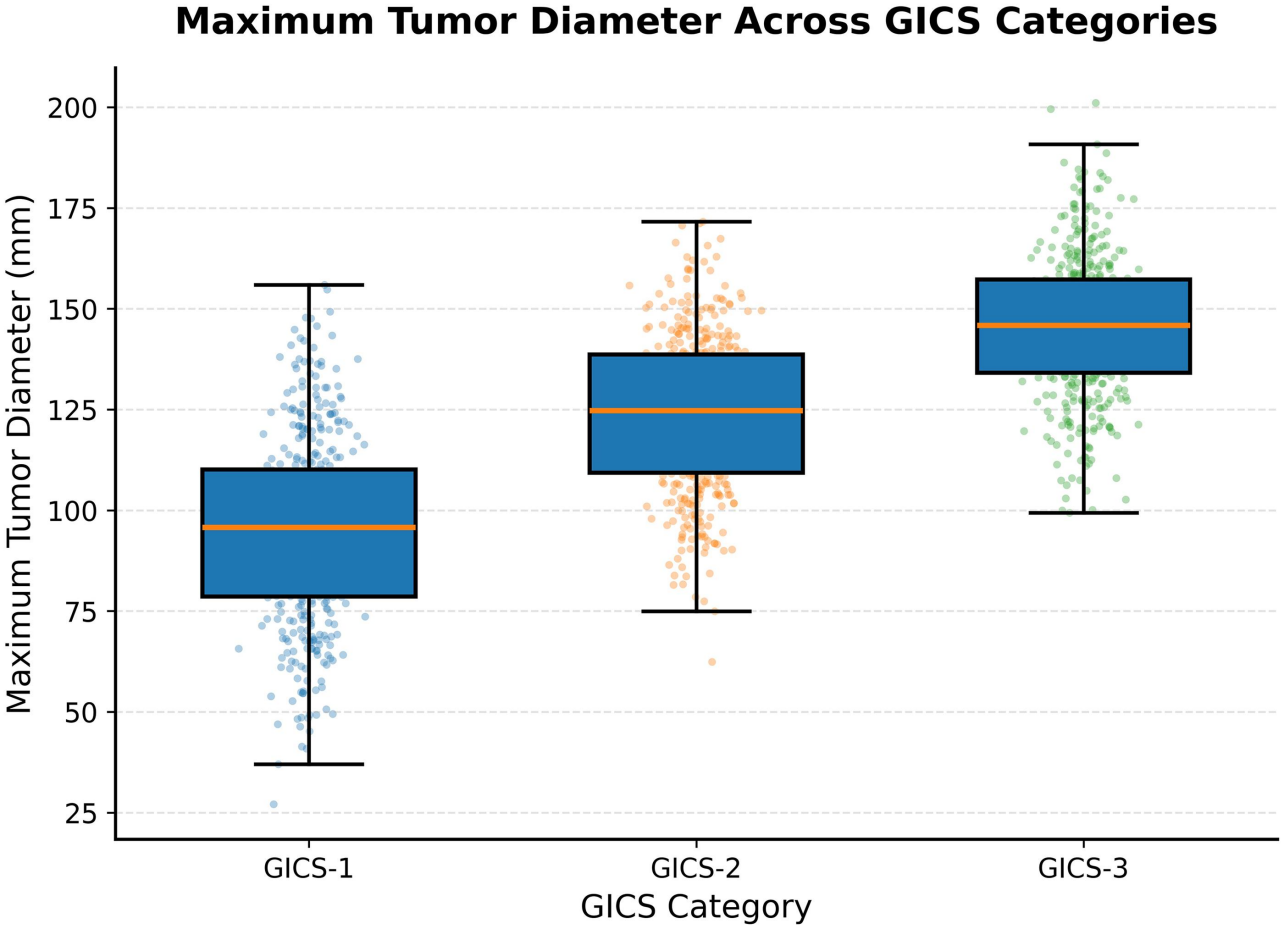
Distribution of maximum tumor diameter across GICS-defined categories.

**Figure 5:**
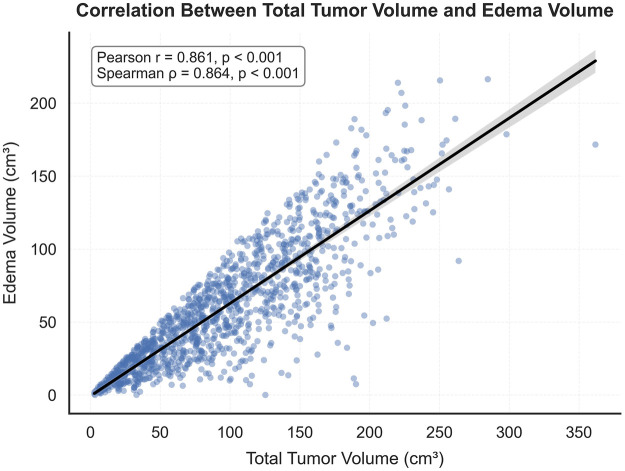
Correlation between total tumor volume and edema volume.

- The distribution of total tumor volume across GICS-defined categories is shown in Figure 3.
- The distribution of maximum tumor diameter across GICS-defined categories is shown in Figure 4.
- The correlation between total tumor volume and edema volume is shown in Figure 5.

## Discussion

This study developed a quantitative MRI-based framework for stratifying glioma cases into imaging-defined complexity categories using standardized segmentation data from the Brain Tumor Segmentation (BraTS) initiative. Stepwise increases in total tumor volume, edema burden, maximum tumor diameter, enhancing tumor volume, and necrotic/non-enhancing core volume were observed across GICS categories. The present analysis suggests that quantitative MRI-derived variables can be systematically organized into reproducible MRI-based stratification groups across a large multicenter glioma dataset.

Radiomics and quantitative MRI analysis are increasingly influencing modern neuro-oncology research. Conventional MRI interpretation often depends on subjective visual assessment of tumor burden, edema, infiltrative spread, and anatomical distortion. Although experienced neuroradiologists and neurosurgeons routinely integrate these features during clinical decision-making, variability between observers may still occur, particularly in large or heterogeneous gliomas.

In contrast, segmentation-based volumetric analysis provides an objective characterization of tumor morphology using reproducible computational methods. Voxel-based analysis also permits more comprehensive evaluation of irregular tumor architecture than conventional two-dimensional measurements obtained from selected axial slices.

Radiomics and computational neuroimaging approaches increasingly rely on standardized imaging datasets capable of supporting reproducible quantitative analysis across institutions and imaging platforms.

Within this context, increasing tumor burden was associated with coordinated variation across multiple MRI-derived variables. Larger tumors were consistently associated with greater edema burden and larger maximum tumor diameter, suggesting broader spatial involvement and increasing anatomical distortion on imaging analysis. These imaging relationships may be relevant from a neurosurgical perspective because extensive edema and larger tumor size frequently contribute to mass effect, ventricular compression, narrowing of operative corridors, and displacement of surrounding white matter pathways.

From a neuro-oncological perspective, extensive peritumoral edema may additionally reflect disruption of surrounding tissue microarchitecture and altered blood–brain barrier integrity. These imaging characteristics frequently contribute to regional anatomical distortion and may complicate operative exposure, tissue dissection, and preservation of adjacent functional structures.

The strong correlation identified between total tumor volume and edema volume further supports the structural coherence of the proposed framework.

Similarly, the increasing maximum tumor diameter across GICS categories suggests greater spatial expansion and radiographic involvement beyond the primary lesion.

Scatterplot analysis demonstrates a strong positive association between total tumor volume and edema burden across the glioma cohort. The observed relationship supports the coordinated increase in radiographic burden across higher GICS categories.

Although these imaging features cannot independently determine operative difficulty, their coordinated distribution across categories supports MRI-based imaging complexity stratification.

Importantly, the practical implications of imaging-defined complexity may also be influenced by the availability of modern intraoperative technologies. Contemporary glioma surgery increasingly incorporates adjuncts such as neuronavigation, intraoperative ultrasound (IoUS), and intraoperative neurophysiological monitoring (IONM), which may enhance surgical orientation, facilitate maximal safe resection, and improve preservation of neurological function.

As a result, the practical surgical complexity of a lesion may vary depending on the technological resources available in a given neurosurgical environment.

Although the current GICS framework was intentionally designed as an MRI-based stratification model, future iterations may benefit from integrating technological adjuncts and operative planning variables alongside imaging characteristics to provide a more comprehensive assessment of glioma complexity and the potential for maximal safe resection (10).

Previous studies have also explored the relationship between imaging characteristics and glioma surgical complexity. Ganau et al. proposed a grading system for high-grade glioma surgery that incorporates eloquent cortical involvement, ventricular relationships, vascular anatomy, and proximity to white matter tracts. Marcus et al. similarly demonstrated that preoperative MRI features may help predict surgical outcomes in patients with glioblastoma. Unlike these clinically oriented systems, the present study focused primarily on reproducible segmentation-derived imaging features obtained from standardized multicenter datasets.

This distinction is important because many previously reported glioma grading systems were developed primarily to estimate operative complexity or predict postoperative outcomes using anatomical and clinical variables. In contrast, the current framework was designed to evaluate reproducible MRI-derived volumetric relationships across a harmonized multicenter dataset.

The use of standardized segmentation masks and preprocessing pipelines additionally reduced observer-dependent variability commonly associated with qualitative MRI interpretation.

It is important to emphasize that the primary objective of the present study was not to demonstrate superiority over existing volumetric assessment methods, but rather to establish a reproducible organizational framework capable of categorizing glioma imaging burden using standardized segmentation-derived MRI variables. The GICS model therefore represents an initial imaging stratification approach designed to facilitate structured quantitative comparisons across large datasets. Future studies will be required to determine whether GICS categories provide incremental value beyond conventional volumetric measurements for operative planning, extent of resection, functional outcomes, molecular characteristics, or survival.

Furthermore, unlike conventional radiological assessment, segmentation-derived volumetric analysis permits quantitative characterization of irregular tumor morphology and heterogeneous infiltrative patterns that may not be fully captured using two-dimensional measurements alone.

The increasing integration of radiomics, quantitative imaging analysis, and machine learning within neuro-oncology further highlights the potential importance of reproducible MRI-based stratification approaches for future imaging-oriented research workflows.

This comparison highlights the computational imaging-oriented nature of the GICS framework and further distinguishes it from clinically validated operative grading systems.

An important strength of the current investigation is the use of a large, harmonized imaging cohort from the BraTS initiative. Standardized preprocessing pipelines and expert-annotated segmentation masks enabled consistent extraction of imaging features across more than 1,200 glioma cases while reducing observer-dependent variability. The framework, therefore, reflects a computational imaging approach designed to support future radiomics-oriented neuro-oncology research.

Standardized MRI-derived stratification systems may also contribute to future visualization-assisted neurosurgical workflows. Emerging technologies, including augmented reality neuronavigation, mixed reality surgical simulation, three-dimensional reconstruction, and AI-assisted operative planning, increasingly rely on structured imaging datasets that enable reproducible computational analysis. Frameworks such as GICS may support future studies exploring imaging-guided surgical planning, radiomics-assisted stratification, and advanced visualization workflows within neuro-oncology.

The MRI variables included in the GICS framework reflect different aspects of glioma morphology, radiographic heterogeneity, and spatial anatomical involvement. Total tumor volume primarily reflects overall lesion burden and anatomical occupation, whereas edema volume may indicate disruption of surrounding tissue and regional mass effect. Enhancing tumor volume reflects contrast-enhancing biologically active tumor regions, while necrotic/non-enhancing core volume may correspond to intratumoral degeneration and increasing radiographic heterogeneity. Maximum tumor diameter provides an estimate of spatial anatomical expansion and infiltrative involvement across surrounding brain structures.

Collectively, these imaging variables support computational characterization of glioma morphology and anatomical burden using standardized volumetric analysis.

Nevertheless, several limitations should be acknowledged. First, the study was retrospective and relied exclusively on publicly available imaging datasets without prospective clinical validation. Second, the framework focused primarily on volumetric imaging features and did not incorporate tractography, vascular anatomy, eloquent cortical involvement, molecular profiling, functional imaging, or postoperative neurological outcomes. Third, the current model was based primarily on total tumor volume stratification rather than a fully weighted composite scoring system. Consequently, the present framework should be interpreted as an organizational imaging classification rather than evidence that total tumor volume alone fully captures the multidimensional nature of glioma complexity.

An additional limitation is that the GICS framework was not directly compared with established response assessment systems such as RANO 2.0. While RANO 2.0 is primarily designed to standardize treatment response assessment and disease progression evaluation, GICS was developed as a preoperative imaging-based framework to stratify glioma surgical complexity. Therefore, the two systems address different clinical objectives. Nevertheless, future investigations may benefit from formal comparative analyses that evaluate the complementary value, performance characteristics, and potential non-inferiority of these approaches for clinical decision-making and outcome prediction.

Finally, no external validation cohort was included, limiting assessment of generalizability across independent clinical populations.

Despite these limitations, the findings demonstrate that standardized quantitative MRI variables can be reproducibly organized into structured glioma imaging categories across large multicenter datasets.

Future investigations should evaluate whether MRI-derived GICS categories correlate with operative findings, extent of resection, postoperative neurological outcomes, molecular tumor characteristics, and long-term survival within prospective clinical cohorts.

Future development of the framework may also benefit from incorporating tractography, anatomical localization, perfusion imaging, diffusion-weighted imaging, and molecular tumor profiling.

## Conclusion

The present study suggests that volumetric MRI features can be organized into reproducible radiographic stratification categories using standardized segmentation datasets from the Brain Tumor Segmentation (BraTS) initiative.

The proposed GICS model represents a preliminary imaging stratification framework intended to characterize relative glioma imaging patterns, rather than a validated operative grading or surgical outcome prediction tool.

Importantly, the present study does not establish clinical superiority over existing volumetric assessment approaches; rather, it provides a reproducible framework for future investigation and validation.

Overall, the findings suggest that standardized volumetric MRI analysis can support reproducible characterization of glioma imaging patterns across multicenter datasets. However, further prospective evaluation and external validation incorporating intraoperative findings, extent of resection, functional outcomes, molecular characteristics, and longitudinal clinical data will be necessary before determining the broader translational and clinical applicability of the proposed model.

## Data Availability

Publicly available datasets were analyzed in this study. The data are available from the Brain Tumor Segmentation (BraTS) initiative via the official repository: https://www.med.upenn.edu/cbica/brats2020/data.html.
